# Neopterin is Associated with Disease Severity and Outcome in Patients with Non-Ischaemic Heart Failure

**DOI:** 10.3390/jcm8122230

**Published:** 2019-12-17

**Authors:** Lukas Lanser, Gerhard Pölzl, Dietmar Fuchs, Günter Weiss, Katharina Kurz

**Affiliations:** 1 Department of Internal Medicine II, Medical University of Innsbruck, 6020 Innsbruck, Austria; lukas.lanser@i-med.ac.at (L.L.); guenter.weiss@i-med.ac.at (G.W.); 2 Department of Internal Medicine III, Medical University of Innsbruck, 6020 Innsbruck, Austria; gerhard.poelzl@tirol-kliniken.at; 3 Division of Biological Chemistry, Biocenter, Medical University of Innsbruck, 6020 Innsbruck, Austria; dietmar.fuchs@i-med.ac.at

**Keywords:** Neopterin, inflammation, heart failure, adverse outcome

## Abstract

Inflammation and immune activation play an important role in the pathogenesis of cardiac remodelling in patients with heart failure. The aim of this study was to assess whether biomarkers of inflammation and immune activation are linked to disease severity and the prognosis of heart failure patients. In 149 patients (65.8% men, median age 49.7 years) with heart failure from nonischaemic cardiomyopathy, the biomarkers neopterin and C-reactive protein were tested at the time of diagnosis. Patients were followed-up for a median of 58 months. During follow-up, nineteen patients died, five had a heart transplantation, two needed a ventricular assistance device, and twenty-one patients had to be hospitalised because of heart failure decompensation. Neopterin concentrations correlated with N-terminal prohormone of brain natriuretic peptide (NT-proBNP) concentrations (rs = 0.399, *p* < 0.001) and rose with higher New York Heart Association (NYHA) class (I: 5.60 nmol/L, II: 6.90 nmol/L, III/IV: 7.80 nmol/L, *p* = 0.033). Higher neopterin levels were predictive for an adverse outcome (death or hospitalisation due to HF decompensation), independently of age and sex and of established predictors in heart failure such as NYHA class, NT-proBNP, estimated glomerular filtration rate (eGFR), and left ventricular ejection fraction (LV-EF) (HR 2.770; 95% CI 1.419–5.407; *p* = 0.003). Patients with a neopterin/eGFR ratio ≥ 0.133 (as a combined marker for immune activation and kidney function) had a more than eightfold increased risk of reaching an endpoint compared to patients with a neopterin/eGFR ratio ≤0.065 (HR 8.380; 95% CI 2.889–24.308; *p* < 0.001). Neopterin is associated with disease severity and is an independent predictor of prognosis in patients with heart failure.

## 1. Introduction

Activation and down-regulation of the immune response are important mechanisms to control tissue damage, initiate the healing process, and to remove dead cells and debris after a harmful stimulus [1]. However, prolonged immune activation promotes local and systemic inflammatory processes, thereby contributing to tissue damage and organ failure over time. This has also been shown in patients with chronic heart failure (CHF) [2], where increased concentrations of circulating cytokines and biomarkers of inflammation were associated with a poor outcome [3,4,5]. Therefore, the balance of physiological and pathological immune activation contributes to heart failure (HF) progression and determines the outcomes of these patients [2]. Immune activation in CHF is driven by several factors: pro-inflammatory cells are found in the failing myocardium itself [3] but systemic immune activation also plays a role [6]. In fact, low-grade immune activation has been established to greatly contribute to atherogenesis [7]. Additionally, circulating endotoxins, which translocate from the intestinal tract into the systemic circulation [8], as well as the hypoxia of body tissues [9,10,11] and central inhibition of the parasympathetic nervous system appear to be involved [6]. 

Elevated parameters of inflammation have been shown to predict an unfavourable clinical course of patients with cardiovascular diseases [12,13,14]. Several studies have demonstrated that the pteridine neopterin is a good prognostic marker for an adverse outcome in patients with clinically inapparent atherosclerosis [12,15], chronic stable angina pectoris [16,17,18], and acute coronary syndrome [19].

Neopterin is produced by activated monocytes, macrophages and dendritic cells (DCs) upon stimulation with interferon gamma (IFN-γ). Therefore, neopterin levels reflect the extent of T-helper cell type 1 (Th1) immune activation. Monocytes stimulated by IFN-γ also produce reactive oxygen species (ROS) [20] concomitantly with neopterin, thus inducing oxidative stress, which also plays a key role in the progress of HF [21,22,23]. Neopterin was demonstrated to correlate with cardiac dysfunction following cardiac surgery [24] and cardiac remodelling in patients with CHF [25]. In addition, neopterin concentrations correlated with the severity of heart failure in patients with preserved ejection fraction (HFpEF) and the probability of future cardiovascular events [26]. 

The aim of this study was to assess the relationship between the inflammatory biomarkers, C-reactive protein (CRP), neopterin, and disease severity, as well as to evaluate the predictive value of these parameters for the outcome of HF patients with nonischaemic cardiomyopathy (CMP).

## 2. Experimental Section

### 2.1. Study Population

We retrospectively analysed the data of 475 caucasian patients with HF caused by nonischaemic CMP. Patients with more than mild-to-moderate valve disease as well as ischaemic cardiomyopathy were not included in the study, since there are studies describing significant differences in immune activation between patients with ischemic and non-ischemic cardiomyopathy [27]. At our department, specific investigations such as echocardiography, coronary angiography (CAG), right heart catheterization, and endomyocardial biopsy (EMB) were performed in patients with nonischaemic CAG. These investigations took place between 2009 and 2014 over the course of an elective hospitalisation, and only patients with compensated HF were investigated. All patients were diagnosed and treated according to prevailing guidelines at the cardiology department at Innsbruck University Hospital. Data of all HF patients with available neopterin and C-reactive protein concentrations (n = 149) were analysed. The final study population consisted of 98 men and 51 women. The study conformed to the ethical principles outlined in the Declaration of Helsinki and was approved by the ethics committee of the Innsbruck Medical University (ID of the ethical votum: UN4280, session number 298/4.11). All patients gave written informed consent to participate in this study.

### 2.2. Follow-Up Analysis

Patients were followed up until May 2017. For the outcome analysis, we defined the event-free survival as time between invasive diagnosis and laboratory testing, and the occurrence of the combined endpoint. Components of the combined event were death or hospitalisation for cardiac decompensation, whatever came first. Information about patients’ events was obtained from the clinical information system (KIS), the local mortality registry, from the patients’ relatives or from the patients themselves. 

### 2.3. Measurements

Blood samples were taken from all patients at their first hospitalisation and stored at −80°C. Concentrations of all laboratory variables were measured at the central laboratory of the Innsbruck University Hospital, which undergoes regular internal and external quality control and evaluation. Neopterin was measured by an enzyme-linked immunosorbent assay (IBL International GmbH, Hamburg, Germany). C-reactive protein (CRP) was detected with an immunoturbidimetry test (Roche, Mannheim, Germany). In order to estimate the glomerular filtration rate (eGFR), we used the IDMS-traceable MDRD study equation (eGFR(mL/min/1.73 m2) = 175 × (serum creatinine) − 1.154 × age − 0.203 (×0.742 if female)). 

Hemodynamic parameters were measured in the course of a right and left heart catheterisation, while the left ventricular ejection fraction (LV-EF) was measured during an echocardiography. 

### 2.4. Statistical Analysis

Quantitative variables are presented as medians (25th, 75th percentile) because there was no Gaussian distribution given. Categorical variables are presented as prevalence and percentage. The Kolmogorov-Smirnov test was used to evaluate the normal distribution of the measured data. To test for differences between two or more groups, Mann-Whitney-U test (two unpaired groups), Kruskal-Wallis test (more than two unpaired groups) and Pearson chi-square test were used. Spearman rank correlation was used to assess cross-sectional relations between neopterin, HF severity and kidney function. We used proportional hazard regression analysis to analyse the potential risk factors for an adverse outcome and logarithmised parameters that showed a skewed distribution. All tests used were two-tailed and *p*-values < 0.05 were considered as statistically significant. The statistical analysis was performed with SPSS Statistics Version 24.0 for Macintosh (IBM Corporation, Armonk, NY, USA).

## 3. Results

Demographic and clinical characteristics, laboratory measurements, and haemodynamic parameters of the whole population and separately for patients with and without an event within five years are depicted in Table 1.

The percentage of patients with reduced LV-EF <40% was 63.4% (66.3% of men, 58.0% of women, *p* = 0.323). Reduced kidney function (eGFR ≤ 60 mL/min/1.73m^2^) was found in 40 patients (26.8%) but only seven of them (4.7%) were presented with advanced renal insufficiency (eGFR ≤ 45 mL/min/1.73m^2^). 

### 3.1. Inflammation Correlates With HF Severity and Cardiac Function

Inflammatory parameters (CRP and/or neopterin) were elevated in 72 patients (48.3%). Out of these, 25 patients (16.8%) showed elevated CRP concentrations (>0.5 mg/L), 27 patients (18.1%) elevated neopterin concentrations (>8.7 nmol/L), and 20 patients (13.4%) showed both elevated CRP and neopterin concentrations. 

**Figure 1:**
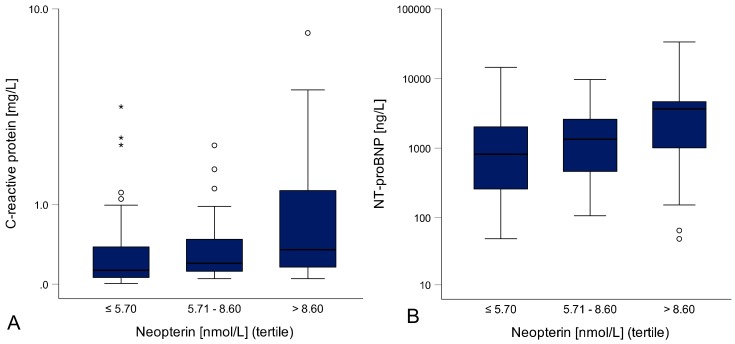

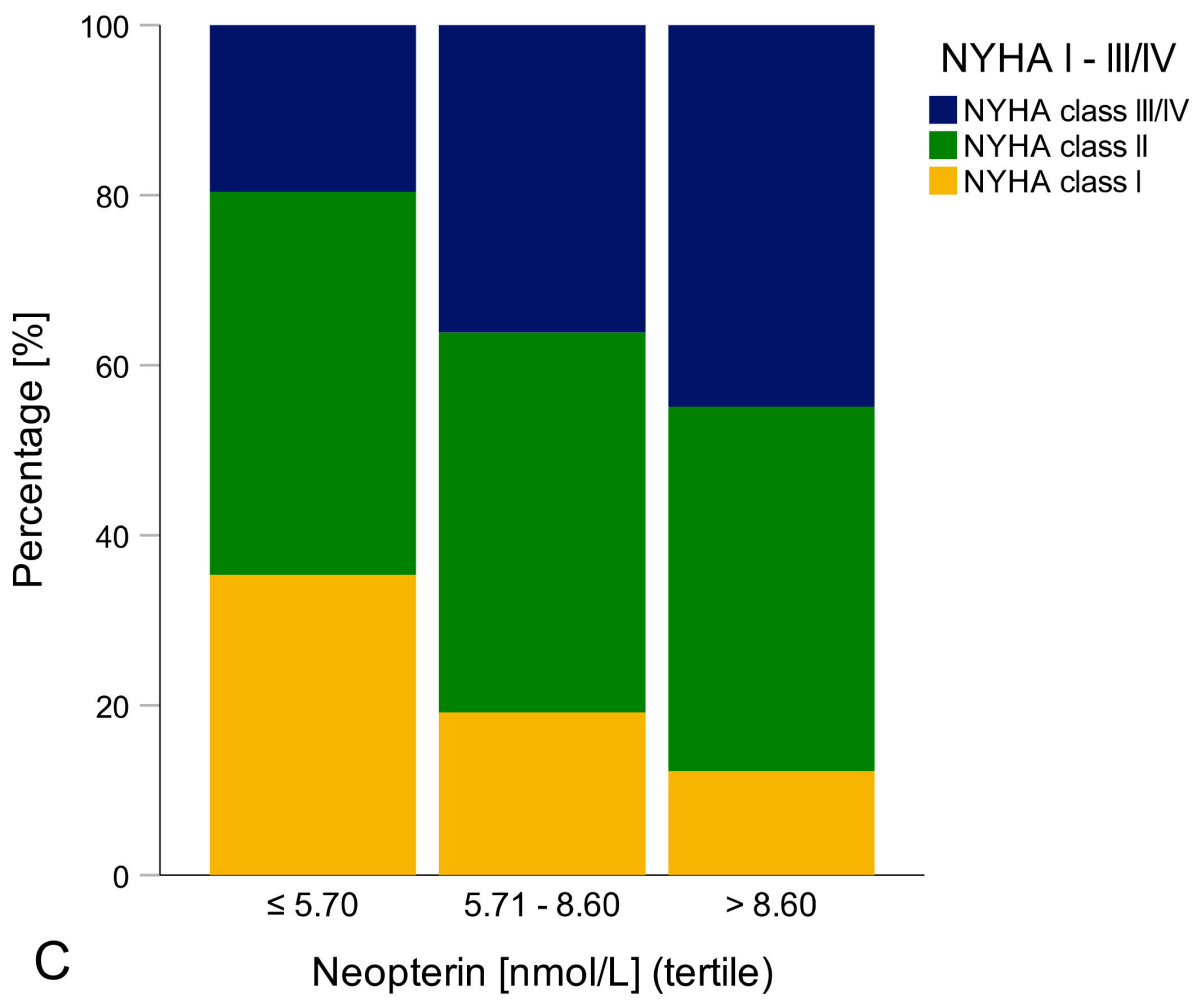
Inflammation and HF severity: Higher neopterin concentrations were associated with higher CRP (**A**) and NT-proBNP concentrations (**B**). Patients with higher neopterin concentrations also had higher NYHA classes (**C**).

Neopterin concentrations were positively correlated with CRP concentrations (rs = 0.343, *p* < 0.001; Figure 1A). Additionally, significant correlations were found between neopterin concentrations and NT-proBNP concentrations (rs = 0.399, *p* < 0.001, Figure 1B), cardiac index (rs = −0.287, *p* = 0.001), right atrial pressure (RAP, rs = 0.170, *p* = 0.043), pulmonary artery mean pressure (mean PAP, rs = 0.227, *p* = 0.007) and pulmonary capillary wedge pressure (PCWP, rs = 0.244, *p* = 0.004) were found. Neopterin progressively increased with higher NYHA class (I: 5.60 nmol/L, II: 6.90 nmol/L, III/IV: 7.80 nmol/L, *p* = 0.033, Figure 1C).

CRP concentrations also correlated significantly with NT-proBNP concentrations (rs = 0.232, *p* = 0.006) and showed a positive dose-response relationship with increasing NYHA class (l: 0.16 mg/L, ll: 0.17 mg/L, lll/lV: 0.25 mg/L, *p* = 0.030).

### 3.2. Neopterin/eGFR Ratio and HF Severity

As patients with reduced eGFR (≤60 mL/min/1.73m^2^) had significantly higher neopterin concentrations than patients with preserved kidney function (8.90 nmol/L vs. 6.00 nmol/L, *p* < 0.001), we adjusted neopterin concentrations for the kidney function and calculated a neopterin/eGFR ratio. Correlation analysis showed a highly significant correlation of the neopterin/eGFR ratio with NT-proBNP concentrations (rs = 0.438, *p* < 0.001), cardiac index (rs = −0.383, *p* < 0.001), right atrial pressure (RAP, rs = 0.172, *p* = 0.041), pulmonary artery mean pressure (mean PAP, rs = 0.281, *p* = 0.001) and pulmonary capillary wedge pressure (PCWP, rs = 0.302, *p* < 0.001). Patients with a higher NYHA class showed a significant higher neopterin/eGFR ratio (l: 0.060, ll: 0.098, lll/lV: 0.131, *p* = 0.003). 

### 3.3. Neopterin/eGFR Ratio and Left Ventricular Ejection Fraction

The LV-EF was reduced (<40%) in 49.7% of our patients (Heart Failure with reduced Ejection Fraction—HFrEF), while 22.1% had a preserved LV-EF ≥ 50% (Heart Failure with preserved Ejection Fraction—HFpEF) and 21.5% a LV-EF between 40%–49.9% (Heart Failure with mid-range Ejection Fraction—HFmrEF). Patients with HFmrEF had the lowest neopterin concentrations (5.35 nmol/L, *p* = 0.021) and the highest eGFR (84.28 mL/min/1.73m2, *p* = 0.003) compared to patients with HFrEF and HFpEF (Appendix A, Table A1). Interestingly enough, neopterin concentrations did not differ significantly between patients with HFrEF and HFpEF (7.00 nmol/L vs. 7.40 nmol/L, *p* = 0.235), while patients with HFpEF had a significantly lower eGFR compared to patients with HFrEF (66.15 mL/min/1.73m2 vs. 76.48 mL/min/1.73m2, *p* = 0.026).

### 3.4. Laboratory Parameters and Event-Free Survival

The median follow-up of patients in this study was 58 months (0–98). A total of 40 patients reached the combined endpoint: 19 patients (12.8%) died and 21 patients (14.1%) were hospitalised for cardiac decompensation. 

Patients with an event within five years had significantly higher neopterin and NT-proBNP concentrations, as well as a higher RAP and were found to have a higher NYHA class, while the cardiac index and eGFR were significantly lower compared to patients without an event. Interestingly enough, CRP concentrations, LV-EF, or age did not differ between patients with or without an event, while patients with an event showed a higher BMI compared to patients with no event (Table 1).

### 3.5. Neopterin is a Predictor for an Adverse Outcome in Patients with HF

**Figure 2:**
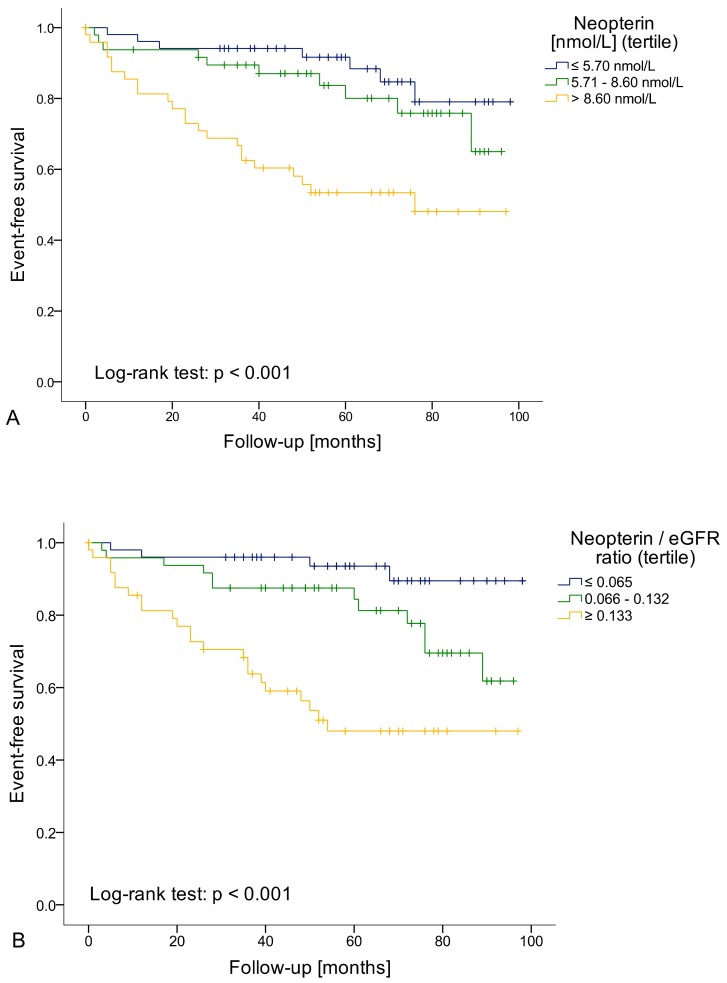
(**A**) Patients with higher neopterin levels (Neopterin > 8.60 nmol/L) had a fourfold higher risk of reaching an endpoint compared to patients within neopterin levels ≤5.70 nmol/L in Cox regression analysis sex-stratified and adjusted for age (*p* = 0.001); (**B**) The same was also true for patients with a higher neopterin/eGFR ratio: Patients with a neopterin/eGFR ratio ≥ 0.133 had a more than eight-fold increased risk of reaching an endpoint compared to patients with a neopterin/eGFR ratio ≤ 0.065 (*p* < 0.001).

Patients with neopterin concentrations >8.60 nmol/L (highest tertile) had a fourfold higher risk of reaching an endpoint compared to patients with neopterin concentrations ≤5.70 nmol/L (lowest tertile) in Cox regression analysis sex-stratified and adjusted for age (HR 4.118; 95% CI 1.727–9.820; *p* = 0.001; Figure 2A). The cumulative five-year event rates for the neopterin tertiles were 8.4% (≤5.70 nmol/L), 20.0% (5.71–8.60 nmol/L) and 46.6% (≥8.61 nmol/L). This was even independent of kidney function since a higher neopterin/eGFR ratio (logarithmised) was also predictive for future adverse events in Cox regression analysis sex-stratified and adjusted for age (Table 2). Patients with a neopterin/eGFR ratio ≥ 0.133 had a more than eightfold increased risk of reaching an endpoint compared to patients with a neopterin/eGFR ratio ≤ 0.065 (HR 8.380; 95% CI 2.889–24.308; *p* < 0.001, Figure 2B).

A multivariate regression model stratified for sex was calculated with neopterin, age, eGFR, NT-proBNP, NYHA functional class, and LV-EF as co-variates that were considered clinically meaningful. Multivariate Cox regression analysis showed that baseline neopterin levels were associated with the combined endpoint, independently of established and widely available predictors of HF such as eGFR, NT-proBNP, NYHA class and LV-EF (Table 2). Neopterin was also an independent predictor for an unfavourable outcome when correcting for co-medications (ACE inhibitor/ARB, beta-blocker, MRA, diuretics or cardiac glycosides) in Cox regression analysis. Patients with diuretics had significantly higher neopterin concentrations than those without (7.50 nmol/L vs. 5.80 nmol/L, *p* = 0.001).

## 4. Discussion

This study demonstrates that serum neopterin concentrations are linked to disease severity and can predict a worse outcome in patients with HF caused by non-ischaemic CMP. We also show that calculation of the neopterin/eGFR ratio is very useful to predict a worse outcome of patients and might be well suited as a “combined” marker for immune activation and decreased kidney function.

Recent studies have proposed a key role of inflammation in the determination of cardiovascular risk [28]. Levels of inflammatory cytokines are elevated in HF patients and related to an adverse outcome [29]. Activation of the immune system following cardiac injury is, per se, a protective (i.e., physiological) mechanism. Several studies have demonstrated that a short-term low-grade expression of stress-activated proinflammatory cytokines within the failing heart has beneficial consequences [30,31,32]. These cytokines induce the upregulation of so-called protective proteins in the heart that are part of the myocardial stress response such as cardiac hypertrophy, cardiac remodeling, and cardiac repair. However, the sustained or excessive expression of proinflammatory cytokines can cause tissue injury, consequently leading to progressive LV dysfunction and adverse LV remodeling [1,5,30]. Accordingly, patients with chronic inflammatory disease including rheumatoid arthritis [33], systemic lupus erythematosus [34] or atopic dermatitis [35] were shown to have an increased cardiovascular risk.

Our data show that higher neopterin concentrations, which originate from activated monocytes and macrophages upon stimulation with the proinflammatory cytokine IFN-γ, are associated with an impaired cardiac function: Elevated neopterin concentrations were found in patients with higher NYHA class, lower cardiac index, and increased NT-proBNP concentrations. 

The association of neopterin concentrations with the combined endpoint was independent of age or sex and established predictors in HF such as NT-proBNP, NYHA class, eGFR, and LV-EF. Interestingly enough, CRP concentrations were not associated with the outcome in our population, although CRP is regarded as a powerful predictor of adverse outcome in cardiovascular disease (CVD) and HF [36]. While CRP is an acute phase protein and synthesised by the liver mainly upon IL-6 [37], neopterin is a more specific marker reflecting the interaction of T-cells (IFN-γ signalling) and monocytes/macrophages within Th1 immune activation. While CRP was shown earlier to be elevated in patients with acute cardiac events (unstable angina pectoris, non-ST-elevation myocardial infarction, or ST-elevation myocardial infarction), neopterin did not differ between these patients, but was predictive for a higher risk of an adverse long-term outcome in patients with coronary artery disease compared to high CRP concentrations [12]. 

There are only few studies in which CRP and neopterin levels were tested in parallel in large populations: In all these trials, neopterin was predictive for an increased cardiovascular mortality, but also for the overall mortality, independent of other established risk factors [12,14,19], and also independent of the acute phase marker CRP. Hazard ratios for adverse outcomes were higher for elevated neopterin concentrations as compared with high CRP concentrations in the LURIC study (patients with different kinds of cardiovascular diseases) [12,38], and neopterin was also predictive of an adverse outcome after adjusting for NT-ProBNP values, while CRP was not. Contrarily, in the HUSK study (population-based study in West Norway) CRP seemed slightly better for the prediction of CVD mortality, while IFN-γ-mediated inflammatory markers (neopterin and tryptophan degradation) better predicted non-CVD mortality [14]. Unfortunately, testing for neopterin is not performed in most routine labs, while the measurement of CRP is easily available everywhere. 

Previous studies have also shown that neopterin, but not CRP, is associated with LV dysfunction [16] and predicts an increased cardiovascular risk [18] in patients with stable angina pectoris. On the other hand, elevated CRP levels are an established cardiovascular risk factor [39], which has also been used recently in the CANTOS trial, which assessed the effect of anti-inflammatory treatment with the monoclonal antibody Canakinumab (targeting interleukin-1β) in patients with prior myocardial infarction and elevated CRP [40]. Canakinumab was very effective in preventing adverse cardiac events and decreasing CRP concentrations in patients, indicating that the downregulation of chronic inflammatory processes is able to improve patient outcomes.

Considering this possible role of immune activation in the pathogenesis of cardiovascular disease, the determination of other inflammatory markers like neopterin appears to be a promising strategy to assess the actual risk of HF patients for a cardiovascular event. In particular, it may also serve as decision-making tool for anti-inflammatory therapy to decide if immune activation is over-whelming or within the normal range. In our population, the neopterin/eGFR ratio was also correlated with all relevant risk markers as well as with HF severity and it was predictive for a worse outcome. Calculation of the neopterin/eGFR ratio might in fact allow an even better risk stratification of patients with HF, as it combines the information from two risk factors (inflammation and decreased renal function). Thus, it would certainly be interesting to investigate the predictive power of this combined marker in future HF trials with a higher number of patients.

Still, it has to be emphasised that there are also other very important factors that contribute importantly to the development of inflammation. Moreover, the interaction between genetic and environmental factors might play a prominent role and significantly modulates inflammatory processes [41]. In patients with HF other mechanisms such as transthyretin amyloidosis or HF with preserved ejection fraction should also be investigated in more detail [42]. Further studies examining the effects of an impaired cholesterol efflux, which is linked to an increased CV risk, might provide interesting new data [43]. Last but not least, the role of diet should be evaluated in more detail in patients with CVD and HF. A very interesting recent study reviewed the impact of diet on inflammation, and in fact, the change of diet might represent a relatively easy and reasonable strategy to reduce the risk of CVD [41].

### Strengths and Limitations

This study shows the clinical potential of neopterin and neopterin/eGFR ratio for the prediction of the course of CHF. Unfortunately, neopterin and CRP were not available in all patients who were initially included in the study, which resulted in a smaller sample size. This must be taken into account when interpreting the results of multivariate Cox regression analysis. The fact that the study was carried out with patients with non-ischemic CMP does not allow for a sweeping generalisation about all HF patients. The collection of event data, including patients questioning themselves and relative driven information, also represents a certain bias. 

## 5. Conclusions

This study indicates that Th_1_ immune activation, reflected by neopterin concentrations, plays a crucial role in the pathogenesis of HF caused by nonischaemic CMP. Neopterin concentrations as well as the neopterin/eGFR ratio are linked to disease severity and are associated with disease progression and an adverse outcome for patients with HF. Further longitudinal studies with a higher number of patients are needed to prove the role of neopterin in HF.

## Figures and Tables

**Table 1:** Patient characteristics.

Variable	Total	No Event *	Event *	Significance
	n = 149	n = 115	n = 34	*p*-Value
	Median (IQR)	Median	Median	
Demographic and clinical characteristics				
Age (years)	49.7 (38.5–61.7)	48.9	51.4	0.074
BMI (kg/m^2^)	24.81 (22.00–27.74)	25.25	23.55	0.025
Heart rate (bpm)	70 (60–82)	70	73	0.220
Diast. BP (mmHg)	80 (70–85)	80	77	0.751
Syst. BP (mmHg)	120 (110–132)	120	115	0.193
Hypertension	45.2%	45.5%	44.1%	0.884
Atrial fibrillation	9.7%	9.7%	9.4%	0.952
NYHA class, overall	-	-	-	0.072
NYHA class l	22.4%	26.3%	9.1%	-
NYHA class ll	44.2%	43.9%	45.5%	-
NYHA class lll/lV	33.3%	29.8%	45.5%	-
Laboratory measurements				
Neopterin (nmol/L)	6.90 (5.00–9.70)	6.50	10.00	<0.001
CRP (mg/L)	0.20 (0.10–0.63)	0.20	0.20	0.966
eGFR (mL/min/1.73m^2^)	74.11 (58.39–90.47)	78.29	65.57	0.001
Neopterin/eGFR ratio	0.097 (0.057–0.148)	0.082	0.162	<0.001
NT-proBNP (ng/L)	1340 (501–3266)	1025	3835	<0.001
Hemodynamics				
LV-EF (%)	37.0 (25.7–49.7)	36.0	46.0	0.052
Cardiac index (L/min/m^2^)	1.93 (1.68–2.45)	2.01	1.78	0.003
mean PAP (mmHg)	26.0 (19.0–33.0)	24.0	32.5	0.001
PCWP (mmHg)	17 (11–25)	15	24	<0.001
RAP (mmHg)	9 (6–12)	8	11	0.003
Medication and Treatment				
ACE inhibitor/ARB	77.7%	79.8%	70.6%	0.256
Beta-blocker	74.8%	78.8%	61.8%	0.045
MRA	34.5%	33.3%	38.2%	0.598
Diuretics	57.8%	52.6%	75.8%	0.018
Cardiac glycosides	2.0%	1.8%	2.9%	0.666
Pacemaker	3.4%	3.5%	2.9%	0.872

Data from 149 patients are presented as medians (interquartile range). (*) Event within five years. Parameters that differed significantly are printed in italic letters. IQR = interquartile range; BMI = body mass index; BP = blood pressure; NYHA = New York Heart Association; CRP = C-reactive protein; eGFR = estimated glomerular filtration rate; NT-proBNP = N-terminal prohormone of brain natriuretic peptide; RAP = right atrial pressure; mean PAP = mean pulmonary artery pressure; PCWP = pulmonary capillary wedge pressure; LV-EF = left ventricular ejection fraction; ACE = angiotensin converting enzyme; ARB = angiotensin II receptor blocker; MRA = mineralocorticoid receptor antagonist.

**Table 2:** Cox regression analysis.

Variable	Univariate Model			Multivariate Model		
	HR	95% CI	*p*-Value	HR	95% CI	*p*-Value
Neopterin (nmol/L) _Ln *	2.874	1.663–4.966	<0.001	2.770	1.419–5.407	0.003
eGFR (mL/min/1.73m^2^) _Ln	0.321	0.174–0.593	<0.001	2.723	0.936–7.926	0.066
NT-proBNP (ng/L) _Ln	1.665	1.253–2.214	<0.001	1.368	0.972–1.926	0.072
NYHA class II vs. I	2.542	0.852–7.578	0.094	3.200	0.830–12.329	0.091
NYHA class III/IV vs. I	3.245	1.070–9.840	0.038	3.126	0.751–13.006	0.117
LV-EF (%) _Ln	2.245	0.989–5.096	0.053	2.884	1.096–7.589	0.032
Neopterin/eGFR ratio _Ln	1.748	1.420–2.152	<0.001			
Cardiac index (L/min/m^2^) _Ln	0.250	0.062–1.008	0.051			
mean PAP (mmHg) _Ln	2.979	1.168–7.599	0.022			
PCWP (mmHg)_Ln	2.453	1.203–5.002	0.014			
RAP (mmHg) _Ln	3.536	1.584–7.894	0.002			
BMI (kg/m^2^) _Ln	0.188	0.032–1.114	0.066			

Univariate and multivariate Cox regression analyses models are adjusted for age and stratified for sex. * Neopterin levels were also adjusted for the eGFR in the univariate model. Variables showing a skewed distribution were logarithmised with the natural logarithm and marked with “_Ln”. HR = hazard ratio; CI = confidence interval; eGFR = estimated glomerular filtration rate; NT-proBNP = N-terminal prohormone of brain natriuretic peptide; NYHA = New York Heart Association; LV-EF = left ventricular ejection fraction; mean PAP = pulmonary artery mean pressure; PCWP = pulmonary capillary wedge pressure; RAP = right atrial pressure; BMI = body mass index.

## Appendix A

**Table A1:** Heart failure classification, demographic and clinical characteristics and laboratory measurements.

Variable	HFrEF, LV-EF < 40%	HFmrEF, LV-EF 40–49.9%	HFpEF, LV-EF ≥ 50%	Sig.
	n = 74	n = 32	n = 33	*p*-Value
	Median (IQR)	Median (IQR)	Median (IQR)	
*Demographic and clinical characteristics*				
Age (years)	46.3 (36.2–55.8)	46.3 (35.3–55.8)	55.6 (48.2–69.2)	0.005
BMI (kg/m^2^)	24.00 (21.60–27.30)	24.76 (22.35–28.05)	24.40 (22.00–28.90)	0.516
Heart rate (bpm)	72 (63–85)	69 (60–79)	70 (60–81)	0.385
Syst. BP (mmHg)	115 (110 – 140)	126 (110–140)	126 (120–150)	0.003
*Laboratory measurements*				
Neopterin (nmol/L)	7.00 (5.20–9.20)	5.35 (3.95–8.10)	7.40 (5.90–11.50)	0.021
CRP (mg/L)	0.24 (0.13–0.63)	0.25 (0.07–0.63)	0.15 (0.10–0.25)	0.120
eGFR (mL/min/1.73m^2^)	76.48 (58.09–94.34)	84.28 (71.12–101.40)	66.15 (54.16–74.23)	0.003
Neopterin/eGFR ratio	0.104 (0.059–0.144)	0.061 (0.046–0.103)	0.126 (0.082–0.190)	0.005
NT-proBNP (ng/L)	2072 (949–3681)	198 (96–745)	1989 (703–4644)	<0.001

Data from 149 patients are presented as medians (interquartile range). Parameters that differed significantly are printed in italic letters. HFrEF = Heart failure with reduced Ejection Fraction; HFmrEF = Heart failure with mid-range Ejection Fraction; HFpEF = Heart Failure with preserved Ejection Fraction; IQR = interquartile range; BMI = body mass index; Syst. BP = systolic blood pressure; CRP = C-reactive protein; eGFR = estimated glomerular filtration rate; NT-proBNP = N-terminal prohormone of brain natriuretic peptide; RAP = right atrial pressure; mean PAP = mean pulmonary artery pressure.
